# New to health sciences librarianship: strategies, tips, and tricks

**DOI:** 10.5195/jmla.2021.1184

**Published:** 2021-04-01

**Authors:** Kelsa Bartley, Jahala Simuel, Jamia Williams

**Affiliations:** 1 k.bartley@med.miami.edu, Assistant Professor, Education and Outreach Librarian, Louis Calder Memorial Library, University of Miami Miller School of Medicine, Miami, FL; 2 jahala.simuel@howard.edu, Medical Librarian, Head of Access Services, Louis Stokes Health Sciences Library, Howard University, Washington, DC; 3 jwilliams@brockport.edu, Health Sciences Librarian, Drake Memorial Library, SUNY Brockport, Brockport, NY

**Keywords:** health sciences librarianship, new librarians, new health sciences librarians, professional development strategies, mentorship

## Abstract

Three new librarians highlight their varied pathways into health sciences librarianship and offer insight into how they are navigating the challenges and successes of being new to the profession. The authors define a new health sciences librarian as a person who has fewer than five years of experience in health sciences librarianship specifically, having either recently graduated from library school or entered the health sciences from another type of librarianship. Jamia Williams speaks about her journey from new MLS graduate to health science librarian; Kelsa Bartley details her transition from library professional to health science librarian; and Jahala Simuel shares her experiences moving from academic librarian to health science librarian. This commentary provides strategies, tips, and tricks that new health sciences librarians may use to hone their craft and explore opportunities for professional development.

## INTRODUCTION

Health care, technology, and education are changing dramatically, so health sciences librarians and libraries must continuously adapt to keep up with the needs of patrons. We must be proactive in recognizing trends in the health sciences, anticipating patron needs, and exploring and assuming new roles to assist our institutions in meeting their missions. By linking career goals and activities to institutional strategic goals for research, patient care, and education, new health sciences librarians will see the need to take a proactive, relevant role by adapting new skills and information.

**Figure 1:**
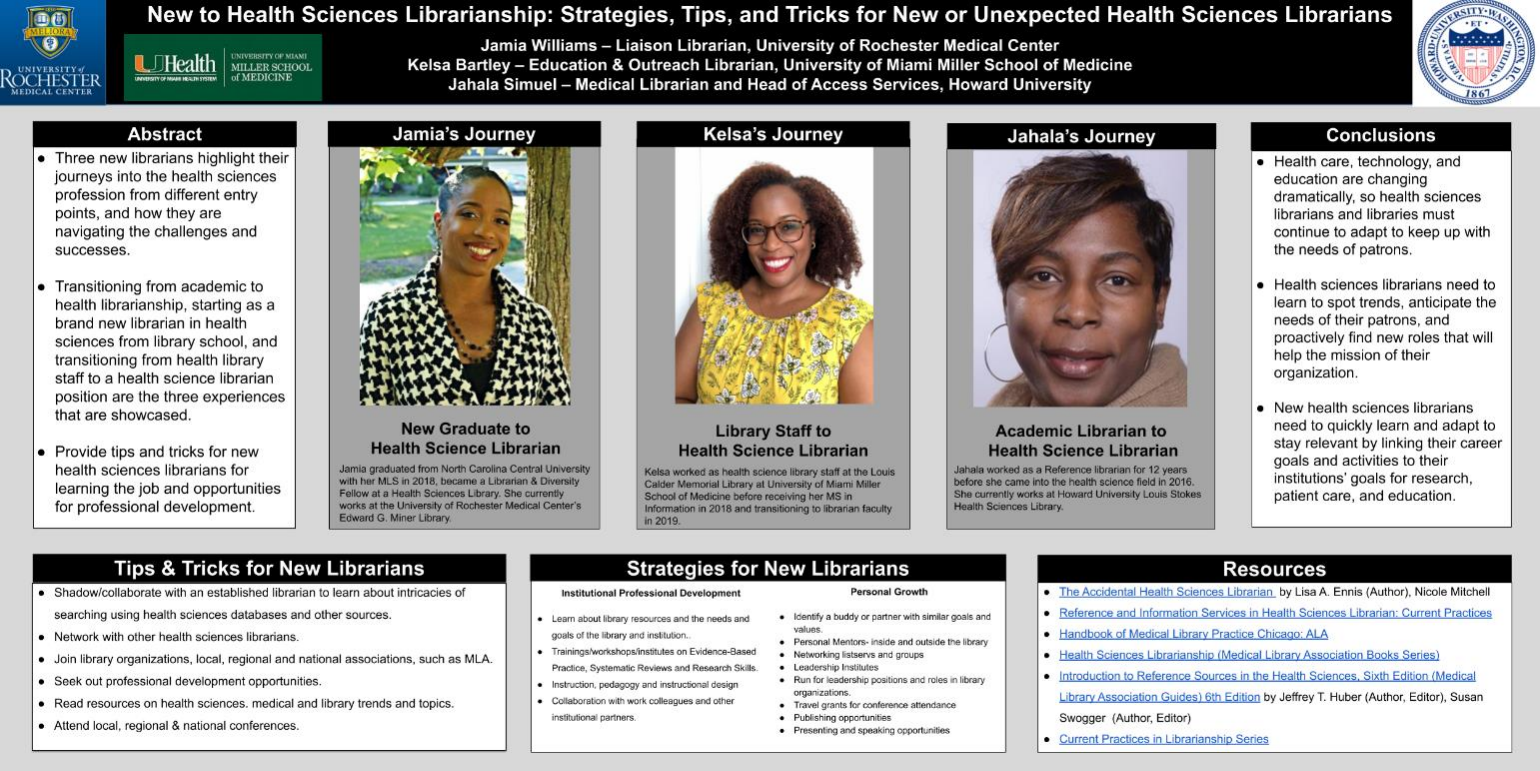
Authors’ poster presented at the 2020 vConference of the Medical Library Association

In this commentary, three new librarians highlight their varied pathways into health sciences librarianship and offer insight into how they are navigating the challenges and successes of being new to the profession. We define a new health sciences librarian as a person with fewer than five years of experience in the profession, having either recently graduated from library school or entered the health sciences from another type of librarianship. Expanding on a poster presented at the 2020 vConference of the Medical Library Association (Figure 1), we discuss strategies, tips, and tricks to help new health sciences librarians learn their jobs and explore opportunities for professional development.

Jamia Williams speaks about her journey from new MLS graduate to health science librarian. She graduated from North Carolina Central University with her MLS in 2018 and became a librarian & diversity fellow at the State University of New York (SUNY) Upstate Medical University's Health Sciences Library. Currently, she works at the SUNY Brockport's Drake Memorial Library.

Kelsa Bartley details her transition to health sciences librarianship. She worked as a library professional at the Louis Calder Memorial Library at the University of Miami Miller School of Medicine before receiving her MS in Information in 2018 and transitioning to the library faculty in 2019.

Jahala Simuel shares some of her experiences and motivations for moving from academic librarianship to health sciences librarianship. She worked as an academic reference librarian for 12 years at Shaw University in Raleigh, North Carolina, before joining the Howard University Louis Stokes Health Sciences Library in 2016.

## JAMIA’S JOURNEY

My journey into librarianship was not traditional. As an undergraduate, I considered three career paths: social worker, lawyer, or librarian. After receiving my undergraduate degree in history from the State University of New York at Brockport, I worked for many years in customer service.

Eventually, I decided to pursue the human services field, with the goal of becoming a case manager. I chose this route because I liked advocating for people and supporting them in their life goals. After five years of hard work and determination, I realized my goal of becoming a case manager. However, throughout my career in the human services field, the idea of becoming a librarian never left me. Even though I did not know any Black librarians, I knew I still wanted to pursue a career in librarianship. I was drawn to this field because I like conducting research and supporting people in their research endeavors. However, I knew that my career change had to be well thought out and planned, since I did not have the liberty of wasting any more time and resources trying to figure it out.

In 2015, I began to pursue my interest in librarianship. Through the Rochester Regional Library Council, I was able to set up shadow experiences with an archivist and a school librarian. Even though these two experiences were vastly different, they reaffirmed my desire to become a librarian. Although my interests were in academic librarianship, special collections, and archives, I was open to other areas as well. My pursuit became a reality in May 2018 when I received an MLS degree from North Carolina Central University, the only historically Black college and university (HBCU) currently awarding a master's degree in library science.

While pursuing my graduate degree, I noticed a staggering and glaring lack of diversity in librarianship. This awareness prompted me to put my burgeoning searching skills to the test to learn more about how the library profession was remedying its lack of diversity. One remedy I discovered in academic librarianship was the creation of diversity residencies and fellowships. These programs are offered to recent MLIS or MLS graduates, usually at an academic library, for a two-year term [1]. The program may or may not have rotations, depending on the institution. I was so happy to discover that two institutions near me had started a diversity residency program. I applied to both, and was hired as the diversity fellow at the Health Sciences Library at the SUNY at Upstate Medical University.

My diversity fellowship was a great opportunity and launching pad for my career; however, it did not equip me with the core skills needed to progress in the field. After self-reflection and discussion with other health sciences librarians, this gap in my knowledge became apparent to me. Therefore, I began to join more affinity groups to get the mentorship that I needed. One of those affinity groups is the African American Medical Librarians Alliance (AAMLA). Being a part of AAMLA has provided mentorship and leadership opportunities for me while keeping me steady on my journey within health sciences librarianship. In addition, I was glad that my next position, as a liaison librarian at the University of Rochester's Medical Center's Edward G. Miner Library, was for an early-career librarian. The support and mentorship that I received from my colleagues were pivotal to my career. They were willing to let me shadow them and ask questions. The work culture embraced trying new things and making mistakes without penalty.

Currently, I am a health sciences librarian at SUNY Brockport's Drake Memorial Library, serving as the liaison to the following academic programs: African & African American Studies, Biology, Health Science/Healthcare Studies, McNair Program, and Nursing.

## KELSA'S JOURNEY

My journey to becoming a librarian began unexpectedly—an intriguing job interview at a library changed my career path forever. In 2010, I interviewed for a photography position in the Calder Medical Library's Biomedical Communications Department at the University of Miami. I had recently graduated with a bachelor's degree in photography from Barry University in Miami Shores, Florida. Prior to that interview, I was focused on becoming a photographer, but I was thrown off guard at the interview when the library director suggested I apply for another open library position instead. She pointed out that I could put the customer service skills I had honed previously as a flight attendant and in other customer service jobs to good use in the library. She also encouraged me to seriously consider becoming a librarian. This was during the recession of the late 2000s, when jobs in photography were scarce. The promise of a great salary and benefits at a university was too good to turn down. I swiftly pivoted from pursuing a full-time photography career and began my library career as manager of library services in the Access Services Department.

A short time after I began working at Calder, I had to move back to Trinidad and Tobago, my birthplace, for personal reasons. I found a job as a photography cataloging assistant and worked for two years in a government media library. It was in this position that I realized how my creative skills could potentially have real value in a library environment. When I returned to the United States, I contacted former colleagues at the University of Miami to inquire about library employment opportunities and was hired as manager of library services in the Reference and Education Department. Although I assisted librarians in this role, I was offered many opportunities to teach, develop content for classes and resource guides, and train for new skills. I used my creative talents to assume leadership for developing and managing social media and other avenues for marketing library resources.

Encouraged by my colleagues and supervisors to formally pursue librarianship as a career, I graduated with an MS in information from Florida State University in December 2018 and was promoted to education and outreach librarian in September 2019. It took me 10 years to get to this point. I continue to grow and expand as a librarian by being in a constant state of learning and remaining open to new and unexpected opportunities—just as I was when my library journey began.

## JAHALA’S JOURNEY

I earned a bachelor of science degree from Saint Augustine's College and MIS and MLS degrees from North Carolina Central University. Librarianship is a second career for me; my first was as a computer teacher at a middle school. I started my library journey in November 2004, working as a reference librarian at a four-year university. By my sixth year in this position, I had begun to ponder the type of library (public, school, or health sciences) in which I wanted to continue working. Being a reference librarian had become repetitive; I was doing the same things, such as instruction and information literacy. I wanted to explore other facets of librarianship, but as the saying goes, it is hard for academic librarians to go into public librarianship because academic librarians are not used to dealing with children, younger teenagers, or homeless people. I was unable to find a job in public libraries, yet I met many people at conferences who worked in public libraries and had held multiple positions due to career growth and advancement in public librarianship.

What intrigued me about health sciences was my desire to become more involved with consumer health, health literacy, health disparities, and nutrition. I came into health sciences librarianship in 2016, when I got a job at the Howard University Health Sciences Library, and I have found that it is different from my previous role because I am doing more research with clinical databases, such as PubMed, Cochrane, and Ovid. Also, I deal with graduate students, doctors, residents, and dental students rather than dealing strictly with undergraduate students. Some tips that helped me in my health sciences journey include finding a mentor, joining the Medical Library Association (MLA) and the Mid-Atlantic Chapter of the MLA (MACMLA), networking, attending conferences, and reading *The Accidental Health Sciences Librarian*. As a health sciences librarian, learning is continuous. If your library has limited funding to send you to conferences or workshops, apply for scholarships or invest in yourself.

## TIPS AND TRICKS FOR NEW LIBRARIANS

We have found the following tips and tricks to be the most useful for new librarians. The first tip we suggest is to shadow and/or collaborate with an established librarian to learn about the intricacies of searching health sciences databases and other sources. Building a connection with an experienced colleague is helpful for your continued growth and can lead to an expanded professional network as your colleague connects you with other librarians. This leads us to our next tip, which is networking with other health sciences librarians. Networking is vital to your professional development and is important when you have a question or concern about topics like instruction, literature searching, or database usage. Taking advantage of the variety of different skills and information that your colleagues have to offer is vital to your professional development.

Another tip we would offer is to join local, regional, and national library associations such as the MLA, which has 42 caucuses, or one of its 13 regional chapters. Membership in such associations is an ideal form of engagement for new librarians. Your membership in these organizations will provide multiple options for professional development opportunities, which is our next tip. This is your opportunity to find your place in the various committees or caucuses within an organization. These organizations provide multiple options and opportunities for professional development. For instance, we are deeply embedded in the work of AAMLA caucus and New Members caucus of the MLA.

One of the tenets of librarianship is lifelong learning, which professional development provides. Many of our training opportunities are available online, which is helpful for institutions with minimal professional development budgets. There are resources available on health sciences, medical, and library trends and topics. If your institution conducts Grand Rounds, we suggest you attend them. Grand Rounds are where medical providers discuss their latest research endeavors, which can provide context for the many health topics that you will encounter. Some Grand Rounds occur virtually and are recorded, so if you are not able to attend at that date and time, you can watch it later. Attending professional webinars and reading publications like *MLA Connect*, the *Journal of the Medical Library Association* (JMLA), *The Journal of Academic Librarianship*, and *In the Library with the Lead Pipe* are additional ways you can stay current on library topics and trends.

Our last tip is to attend local, regional, and national conferences. Attending conferences is where you will be able to network, get training, and present on your scholarship. Again, if your institution's professional development budget is not adequate for attending conferences, we suggest applying for scholarships or grants. There are a variety of scholarships and grants from which to choose. Create a spreadsheet with the application deadlines and their criteria to help you keep track of them.

## STRATEGIES FOR CAREER ADVANCEMENT FOR NEW LIBRARIANS

### Institutional professional development

Starting a job as a new librarian can be daunting no matter your level of prior expertise, and being new to the health sciences professions can be particularly scary. However, learning about your library and institution as a whole is a great first step in starting to navigate a new position and career.

Ask your director, supervisor, work colleagues, and yourself these questions:

- What library resources and services are used the most? Do you need training or a refresher on them?
- How do your current knowledge and skills fit into the immediate and long-term goals of the department, library, and institution?
- What skills or training do you need to edify to perform your job optimally?
- Are there any gaps in knowledge or activity in the library that need to be filled? Do you have, or are you interested in developing skills in any of these areas?
- Are there any internal or external committees or groups that you should be a part of or have contact with to enhance your job experience and performance?
- What are your immediate and long-term goals for your career? Are they aligned with those of the library and the institution?

Asking these questions can help you figure out which associations, conferences, departments, groups, programs, projects, and training you may need to be a part of to develop the skills and knowledge for your immediate role and, ultimately, your career. You may also be able to find a niche in an area of need for the library and the community served by the library.

For instance, PubMed for Librarians is a very helpful and essential go-to resource for health sciences database training. Subscribe to the Network of the National Library of Medicine (NNLM) via email to receive training notifications [2]. You will be updated on the latest information and expand your knowledge in other areas of health sciences librarianship. Attending training programs, workshops, or institutes on evidence-based practice, systematic reviews, and research skills is highly recommended for achieving success as a health sciences librarian. You should also consider coursework in instruction, pedagogy, and instructional design, especially if these are a major part of your job function.

Finally, be sure to foster collaboration and relationships with work colleagues and other institutional partners. Working with others on projects, papers, and activities within your local network should positively enhance your work experience, help build relationships, and create opportunities for advancement in your career.

### Self-directed professional development

Developing your strengths and talents using resources outside your institution can be just as important as the skills you develop at work. You are unlikely to get everything you need to grow in the profession from within your library or your institution. Being motivated to seek out resources from various sources, on your own, will be essential to your success.

Find at least one librarian partner or “buddy” at a similar career stage with similar goals and values as yours. Establishing and fostering deep connections with librarian colleagues from comparable backgrounds, at similar life stages, or who dabble in the same hobbies can be professionally and personally motivating and fulfilling [3]. Connecting with someone or a group of people who understand “library life” and can share the triumphs and challenges of being an early-career librarian can make the difference between an engaging career filled with encouragement, opportunities, and support or one filled with monotony, boredom, and isolation.

Professional and personal mentors, both inside and outside the workplace, are a necessity to advancing in any career. Find librarian and non-librarian peers and colleagues in various capacities and levels of leadership to help guide you along the way. Remember that mentoring is a two-way street, and be prepared to be a mentor to your mentors also. We all have valuable experiences we can share with and learn from each other, no matter what level of librarianship we have reached.

Seek out opportunities for leadership, scholarly advancement, and professional and personal growth.

- Apply for publishing opportunities on topics that interest you, no matter the type of institution at which you work.
  - Having published works of any kind—scholarly book chapters, peer-reviewed or other journal articles, acknowledgments on research articles or systematic reviews, blog posts, and other media—can help propel you to your next library opportunity, job, or leadership role.
  - You can be an editor or peer reviewer of works by librarians and researchers on a topic or trend in which you have expertise.
- Present and speak at conferences, meetings, or webinars inside and outside your institution.
  - If your library does not have funds to support your development:
    - Apply for grants and scholarships for conference attendance and continuing education; and
    - Take advantage of funding opportunities available for early-career librarians from numerous library organizations.
- Get involved in, and nominated for, leadership positions and roles in library organizations.
  - Leadership roles within your library may not be readily accessible, only becoming available when someone resigns or retires.
  - Roles in many associations and groups usually have fixed terms for officers, so you can keep volunteering to serve in various roles in an organization.
- Attend leadership and management institutes at various levels of librarianship.

One of the easiest ways to be alerted to opportunities is to sign up for networking and group listservs, newsletters, and social media from library associations, organizations, and groups. Calls for proposals, conference announcements, continuing education programs, funding, and other professional development opportunities are communicated and circulated widely within these forums and channels.

## CONCLUSION

“But the librarian is always learning. Yes, it's a cliché, but it's true: the librarian is always learning, and with that come the stumbles and the light bulb moments.”

*—Alexandria Brackett*

Each of us took different entry points into librarianship and have used various strategies and tips to navigate our experiences as health sciences librarians. We continue to be works in progress as we continue to learn and grow in the profession and as leaders. Success in health sciences librarianship requires adaptability. Your previous work experience can be an asset to your current position, so use it. Your transferable skills are what make you unique. Avoid the “fake it until you make it” mindset. It is okay to not know something and work through it with the library user. Also, it is okay to say, “Let me look into that for you and get back to you.” This helps create a level of trust between you and library users [4]. We hope that the strategies, tips, and tricks we have gained during our journeys will assist you in your career journey to success as a health sciences librarian.

## SOME HELPFUL RESOURCES

*The Accidental Health Sciences Librarian*, by Lisa A. Ennis, Nicole Mitchell

*Introduction to Reference Sources in the Health Sciences, Sixth Edition* (Medical Library Association Guides), by Jeffrey T. Huber, Susan Swogger

*Health Sciences Librarianship* (Medical Library Association Books Series), edited by M. Sandra Wood

*Reference and Information Services in Health Sciences Librarian: Current Practices* by Sandra Wood

Current Practice in Librarianship Series

Handbook of Medical Library Practice
